# Mentalizing Makes Parenting Work: A Review about Parental Reflective Functioning and Clinical Interventions to Improve It

**DOI:** 10.3389/fpsyg.2017.00014

**Published:** 2017-01-20

**Authors:** Andrea Camoirano

**Affiliations:** Associazione Psicologia ClinicaGenoa, Italy

**Keywords:** reflective functioning scale, parenting, parental mentalization, child maltreatment, embodied mentalizing

## Abstract

In the last decade several studies have investigated the role of parental reflective functioning (RF), defined as the parental ability to understand his/her child’s mental states, on the child’s development. Herein, a narrative review on parental RF is presented aimed at (1) presenting an overview of the existing empirical studies, (2) pinpointing unrequited questions, and (3) identifying future research directions. Specifically, the current review focused on (a) the impact of parental RF on the quality of caregiving and the child’s attachment security, (b) the effect of parental RF on the child’s emotion regulation and the child’s RF, (c) maternal RF in women with a history of neglect and abuse, (d) the efficacy of mentalization-based clinical interventions, and (e) the recently developed Parental Reflective Questionnaire. The following terms “maternal RF,” “paternal RF,” “parental RF,” “parental mentalization,” “maternal mentalization,” and “paternal mentalization” were searched in titles, abstracts, and main texts using Medline, Web of Science, and Scopus databases. Next, a search in Mendeley was also conducted. Inclusion criteria comprised original articles if they refer to the RF Scale (Fonagy et al., 1998) and were published in an English language, peer-reviewed journal before July, 2016. According to exclusion criteria, dissertations, qualitative or theoretical papers, and chapters in books were not taken into account. The review includes 47 studies that, taken together, supported the notion that higher parental RF was associated with adequate caregiving and the child’s attachment security, whereas low maternal RF was found in mothers whose children suffered from anxiety disorders, impairment in emotion regulation, and externalizing behaviors. In addition, higher parental RF was associated with better mentalizing abilities in children. However, unexpected findings have emerged from the most recent randomized controlled trials that tested the efficacy of mentalization-based interventions in high risk samples of mothers, raising questions about the suitability of the verbal measures in capturing the mentalizing processes at the root of the parental capacity to be adequately responsive to the child’s emotional needs.

## Introduction

### The Development of the Construct of Parental Reflective Functioning

The construct of reflective functioning emerged more than 20 years ago in an area of psychoanalysis close to the attachment theory that is essentially concerned with the intergenerational transmission of attachment security. In the well-known, classical, prospective study called the “London Parent-Child Project” (Fonagy et al., 1991b), significant concordance was observed between parental and child patterns of attachment. It was hypothesized that the parental capacity to see the child as a psychological entity with a mental experience, as well as to attune with the child’s mental states played a central role in parenting, thus contributing to the development of child attachment security. To test their hypothesis, Fonagy et al. (1991a) developed a scale to assess the parent’s capacity to understand mental states. Initially it was referred to as the “Reflective Self-Function Scale” to indicate the capacity of the Self to recognize and to reflect upon one’s own mental experience, including feelings, thoughts, desires and beliefs, being able to construct representations of one’s own psychic life as well as being aware of the interpersonal implications of the mental states. The Reflective Self-Function Scale was initially desiged to be used in the Adult Attachment Interview (AAI; George et al., 1985) to detect markers of the ability to understand one’s own and others’ mental states while recalling childhood experiences with the attachment figures. It was based on the hypothesis that the capacity to make sense of one’s own personal history in terms of mental states could promote attachment security, which in turn enables the parent to transmit it to her/his child. In line with this hypothesis, findings from the London Parent-Child Project (Fonagy et al., 1991a) showed a strong correlation between the capacity to reflect upon one’s own history and the child’s attachment security. Fonagy and co-workers assumed that the development of the ability to understand one’s own as well as others’ mental states originates from an early parent-child relationship in which the child experiences the caregiver as being able to recognize his/her mental states. Thus, the maternal ability to understand her child’s mental states would be crucial to allow him/her in turn to develop the same capacity.

A revision of the Reflective Self-Function Scale led to the currently used version, which was re-named the Reflective Functioning Scale (RFS; Fonagy et al., 1998) in order to underline that self-reflection is only one facet of the concept which has indeed essentially interpersonal origins and expressions. As stated above, the RFS was initially applied to the AAI transcripts in which some questions were identified as “demand questions” because they require the interviewee to reveal his/her ability to reflect upon their own experiences in terms of mental states (e.g., “Why did your parents behave as they did during your childhood?, Do you think your childhood experiences have an influence on who you are today?), while other AAI questions were considered “permit questions” in that they only allow reflective functioning (e.g., What did you do when you were upset as a child?). According to the manualized guidelines, four markers indicate evidence of reflective functioning, namely “Awareness of the nature of mental states,” “The explicit effort to tease out mental states underlying behavior,” “Recognizing developmental aspects of mental states,” and “Mental states in relation to the interviewer.” An overall score is assigned to the AAI transcript ranging from -1 (Negative RF, i.e., rejected, bizarre, unintegrated or inappropriate RF) to 9 (exceptional RF).

Surprisingly, despite the noteworthy findings from the previous studies, no other studies on parenting using the RFS were published until 2005, while RFS was extensively used in several published papers regarding other psychological fields such as psychopathology and psychotherapy (for a review, Katznelson, 2014). A special section of Attachment and Human Development, the official journal of the Society for Emotion and Attachment Studies (SEAS), was dedicated to parental reflective functioning in 2005. It focused on the mother’s ability to mentalize about her child, not on her ability to mentalize her past attachment experiences. A new measure of RF, called PDI-RF (Slade et al., 2004) was developed by applying the RFS to the Parent Development Interview (PDI; Aber et al., 1985, Unpublished), an interview that was designed to specifically evaluate the parental mental representations of the child, as well as of herself/himself as a parent. All the PDI questions allow reflective functioning, whereas some questions, such as “When your child is upset, what does s/he do and how does that make you feel?,” require the interviewee to consider mental states, thus eliciting a mentalizing stance. RFS was also applied to the Pregnancy Interview (PI; Slade et al., 2007, Unpublished), an adaptation of the PDI, which was developed to evaluate the mental representation of the mother about herself as being pregnant and about the fetus, and her expectations of her future as a parent. PI-RF was designed to assess the future mother’s ability to mentalize her own emotional experience as well as her proclivity to hold the child in her mind. Some researchers (Schechter et al., 2008) applied the RFS to the Working Model of the Child Interview (WMCI; Zeanah et al., 1995–2000, Unpublished), a measure conceptually similar to the PDI. Applying the RFS to measures that were specifically designed to investigate the parental mental representations of the child was considered particularly noteworthy in that it allows researchers and clinicians to assess mentalization in the specific context of the parent-child relationship which, in addition, is an ongoing relationship, in contrast with the past, recalled relationships in the context of the AAI (Slade et al., 2005).

### The Current Review

Although parental mentalization has been operationalized in several ways leading to the development of various measures (e.g., Maternal Insightfulness; MI; Koren-Karie and Oppenheim, 2001, Unpublished; Maternal Mind-mindedness, MMM; Meins et al., 2001, 2002, 2003), we believe that it is necessary to individually investigate the contribution that each operationalization brought to the research in this field because to date, it is not clear whether the different operationalizations tap into the same component of the construct of parental mentalization (Sharp and Fonagy, 2008). In this paper, we specifically reviewed studies in which parental reflective functioning was measured through the RFS, applied to AAI, PDI, PI, and WMCI (which from here on we refer to as AAI-RF, PDI-RF, PI-RF, and WMCI-RF). Thus, we especially focused on the maternal ability to recognize the nature of the mental states as well as on the proclivity to understand one’s own and others’ behavior as prompted by mental states in the context of autobiographical narratives concerning attachment in family relationships. To date, and to the best of our knowledge, no reviews specifically dedicated to empirical research on parental reflective functioning have ever been published. Thus, a narrative review on parental reflective functioning is presented herein in an effort to (1) present an overview of the existing empirical studies; (2) pinpoint unrequited questions, and (3) identify future research directions. Specifically, the current review focused on (a) the impact of parental reflective functioning on the quality of caregiving and the child’s attachment security, (b) the effects of parental RF on the child’s emotion regulation and the child’s RF, (c) maternal RF in women with a history of neglect and abuse, (d) the efficacy of mentalization-based clinical interventions, and (e) the recently developed Parental Reflective Questionnaire (Luyten et al., 2009, Unpublished).

### Methods

In order to carry out a narrative review on parental reflective functioning, the following terms “maternal reflective functioning,” “paternal reflective functioning,” “parental reflective functioning,” “parental mentalization,” “maternal mentalization,” and “paternal mentalization” were searched in titles, abstracts, and main texts using Medline, Web of Science, and Scopus databases. Next, a search in Mendeley was also conducted. Inclusion criteria comprised original articles if they refer to the RFS (Fonagy et al., 1998) and were published in an English language, peer-reviewed journal before July, 2016. According to exclusion criteria, dissertations, qualitative or theoretical papers, and chapters in books were not taken into account. After applying the exclusion criteria, 158 papers were found to be potentially relevant, the abstracts were then examined, and finally 47 studies which fully met the inclusion criteria were selected for the present review.

## Results

### Parental RF, Quality of Caregiving, and Attachment Security

The first empirical studies using PDI-RF showed that parental RF was closely related to maternal behavior and the child’s attachment security (Grienenberger et al., 2005; Slade et al., 2005). In particular, Slade et al. (2005) found that mothers who were classified as secure on AAI during pregnancy had a higher level of reflective functioning on PDI-RF when the child was 10 months old, and that the children of reflective mothers were more frequently assessed as secure by the Strange Situation procedure (Ainsworth et al., 1978) when they were 14 months old. These findings supported the hypothesis that the maternal coherence of mind in representing her own childhood attachment experiences promotes the development of the ability to consider the child as a psychological agent and makes sense of the child’s behavior in terms of mental states. This is a maternal ability that in turn promotes the child’s attachment security. Conversely, low maternal RF was associated with ambivalent-resistant and disorganized child attachment patterns (Slade et al., 2005) which are important risk-factors for subsequent psychopathology (for a review, Cassidy et al., 2013).

Strong support for the hypothesis according to which maternal PDI-RF and behavior are closely related was also found by Grienenberger et al. (2005) in a low risk sample in which mothers with impaired parental reflective functioning were significantly more often inclined to disrupt affective communication with their children, exhibiting difficulty in regulating their infants’ fear and distress, thus failing to provide them with a feeling of security. It was assumed that low reflective functioning mothers were unable to tolerate and make sense of their children’s painful emotional experience and therefore, that they were not able to help their children to regulate their distress. Schechter et al. (2008) partially replicated the findings by Grienenberger et al. (2005) in a sample of referred mothers who experienced traumatic and violent events prior to the age of 16 years. In their clinical sample, the quality of maternal mental representations on the WMCI was associated both with caregiving behavior and with WMCI-RF, but the latter did not correlate with atypical maternal behavior. They hypothesized that maternal RF might not be directly correlated with the quality of maternal behavior in mothers affected by psychopathology. Nevertheless, further studies found a significant relationship between maternal RF and quality of caregiving even in clinical samples (Borelli et al., 2012; Huth-Bocks et al., 2014; Stacks et al., 2014).

Several other studies offered strong support to the significant relationship between maternal reflective functioning and adequate caregiving. In a low risk sample, Rosenblum et al. (2008) found that parental WMCI-RF predicted maternal mind-minded comments and behavioral sensitivity beyond the contributions of education and depressive symptoms. A more recent study (Huth-Bocks et al., 2014) found that secure mothers (assessed by the Attachment Script Assessment, ASA; Waters and Rodrigues-Doolabh, 2004, Unpublished) from a high risk sample demonstrated higher PDI-RF, and that it was in turn significantly associated with positive parental interactive behavior during play and teaching tasks with 7-month-old children. These findings were replicated in a recent study (Smaling et al., 2016a) that found that mothers with higher parental reflective functioning, as measured during pregnancy, exhibited more positive behavior during free-play, teaching tasks, and the Still Face Paradigm (Tronick et al., 1978) with their 6-month-old children. The significant relationship between maternal PDI-RF and parenting behavior was also found in a recent study (Stacks et al., 2014) on a relatively large and mixed sample consisting of women both with and without a history of childhood maltreatment from diverse socioeconomic backgrounds. In addition, findings by Stacks et al. (2014) showed that maternal reflective functioning was associated with parenting sensitivity which, in turn, was associated with infant attachment security, thus replicating previous findings (Grienenberger et al., 2005). Only Perry et al. (2015) did not find any significant relationship between PI-RF, PDI-RF and maternal emotional availability in a study that, however, had some methodological flaws concerning, above all, sample size and significant delay in follow-up regarding some mother-child dyads.

Thus, taken together, these reviewed studies showed a strong link between maternal attachment security and PDI-RF, which in turn both play a critical role in the child’s attachment security. Most of the studies also supported the hypothesis according to which maternal reflective functioning is associated with a more adequate maternal caregiving behavior, nevertheless, to date, the specific influence that maternal attachment security and maternal PDI-RF have on the child’s attachment security and maternal caregiving behavior does not appear to be well defined. In addition, the review rather surprisingly highlighted a paucity of studies on the relationship between maternal AAI-RF and PDI-RF, and thus it is not clear whether AAI-RF predicts PDI-RF or what the specific impact of AAI-RF and PDI-RF on caregiving behavior is.

Suchman et al. (2010a) investigated the PDI-RF factor structure in order to understand whether a specific factor was associated to the quality of caregiving. Their study, which was carried out on a sample of substance-dependent mothers of toddlers, yielded a two-factor model of parental reflective functioning which indicated that self-mentalization (for example, assessed by the question: “How has having a child changed you?”) and child-mentalization (for example, assessed by asking the parent: “Tell me about a recent time when your child was very upset”) were two distinct, albeit related, dimensions. In contrast to their hypotheses according to which self-mentalization was expected to predict the overall quality of maternal caregiving, and that the ability to mentalize the child would correlate with maternal contingent behavior and child communication, they found that only self-mentalization was associated with maternal contingent behavior. Interestingly, these findings might suggest that in a clinical sample it is the maternal ability to understand her own mental states, associated with the ability to self-regulate the related affective experience that allows the mother to respond adequately to her child’s needs. The two-factor structure of the PDI-RF was confirmed by a later study (Borelli et al., 2016) in a community sample of parents of school-aged children. However, in this study neither of the PDI-RF dimensions correlated with maternal attachment security, and only child-focused RF was associated with children’s attachment security. It should be underlined that in this study maternal attachment security was evaluated by using a self-report measure (Experiences in Close Relationships-Revised, ECR-R; Fraley et al., 2000) related to attachment in romantic relationships, so this finding is not comparable to findings from previous studies that assessed maternal attachment security in the context of AAI transcripts related to childhood attachment experiences. Nevertheless, the replicated finding of the two-factor structure of the PDI-RF seems to be a promising result which should encourage further, more in-depth studies in order to investigate which dimension of parental RF exerts the greatest influence on the child’s attachment security and maternal caregiving behavior.

It is also noteworthy that despite the promising results from the London Parent-Child Project (Fonagy et al., 1991a,b), research on parental reflective functioning focused only on the mentalization of the child and of him/herself as a parent. In fact, only two papers (Arnott and Meins, 2007; Ensink et al., 2016b) replicated the studies conducted by Fonagy et al. (1991a,b) using AAI-RF. Arnott and Meins (2007) found that maternal AAI-RF during pregnancy predicted parental mind-mindedness at 6 months’ post-partum and infant attachment security at 12 months. Ensink et al. (2016b) also found a significant association between mothers’ mentalization regarding their own early childhood attachment relationships and later parenting and infant attachment. In particular, the study revealed that maternal AAI-RF during the pregnancy protected mothers from acting out negative caregiving behaviors (intrusive, aggressive, and withdrawn) which, in turn, were strongly associated with both the child’s disorganization and insecurity of attachment. The importance and the relevance of these studies in which parental RF was assessed before the child’s birth should be highlighted because they reveal how adult reflective functioning, as developed in the context of the earlier relationships with their parents, exerts a crucial influence on caregiving beyond the child’s temperament and other personal features.

In summary, the reviewed studies confirmed that parental RF plays a crucial role in the quality of caregiving and child attachment security. Nevertheless, some issues need to be investigated in greater detail, above all (a) the relationship between AAI-RF and PDI-RF, (b) the specific contribution of maternal attachment security and maternal PDI-RF to the quality of caregiving and the child’s attachment security, and (c) the different impact of the two dimensions of PDI-RF on caregiving behavior.

### Parental RF, Child Emotion Regulation, and Reflective Functioning

To date, only a few studies have investigated the role of parental reflective functioning on child development and psychopathology. They mainly focused on child emotion regulation in early childhood and mentalization in middle childhood as well as in adolescence.

Three studies (Esbjørn et al., 2013; Heron-Delaney et al., 2016; Smaling et al., 2016b) on child emotion regulation focused on anxiety symptoms in school-aged children, infants’ behavior during the Still Face procedure (Tronick et al., 1978), and infants’ aggressive behavior. Esbjørn et al. (2013) found that low maternal AAI-RF (and not low paternal AAI-RF) was a predictor of higher levels of anxiety in a sample of clinically anxious school-aged children referred for psychological treatment. Heron-Delaney et al. (2016) found that preterm infants of high PDI-RF mothers showed the most negative affects as well as more self-soothing behavior during the Still Face procedure, whereas infants whose mothers were rated lower on PDI-RF exhibited the most negative affects during the reunion-episode. It was argued that maternal reflective functioning might promote emotional self-regulation in the child at the time of distress as well as greater trust in maternal responsiveness. Smaling et al. (2016b) demonstrated that young, pregnant, high-risk women with higher PI-RF reported significantly less aggressive behaviors in their children when they were 6, 12, and 20 months old, regardless of the maternal level of sensitivity and intrusiveness, although mothers with both higher PI-RF and no or low intrusive caregiving were more likely not to report difficulties with their children related to the child’s aggressive behaviors.

Some recent studies investigated the relationship between parental reflective functioning and the child’s mentalization in school-aged children and adolescents. It was observed that maternal AAI-RF predicted the children’s mentalization beyond maternal attachment security (Rosso et al., 2015), correlated with children’s mental state talk (Scopesi et al., 2015), and was associated to the children’s reflective functioning regarding themselves in a sample of school-aged children with a history of abuse (Ensink et al., 2015). Furthermore, PDI-RF correlated positively with child reflective functioning and inversely with child externalizing behaviors (Ensink et al., 2016a). In a rare study which also investigated the role of paternal reflective functioning, Benbassat and Priel (2012) found that both maternal and paternal AAI-RF significantly correlated with the children’s reflective functioning in a community sample of adolescents aged 14–18.

Taken together, the results from these studies offer strong support to the hypothesis according to which maternal mentalization ability is truly crucial in promoting the child’s ability to develop emotion regulation, especially regarding painful affects (Fonagy et al., 2002).

### Maternal RF in Mothers with a History of Neglect and Abuse

Maternal RF was extensively investigated in mothers with a history of neglect and abuse to shed light on the intergenerational transmission of abusive parenting and to design clinical interventions to bring the cycle of abuse to an end. It was assumed that a well-developed ability to mentalize one’s own past painful abusive experiences might make the parent equipped to be sensitive to the risk of engaging in painful and frightening interactions with his/her own child (Allen, 2013). In their pivotal work, Fonagy et al. (1993) argued that the subject’s background does not predict *per se* negative caregiving. Rather, they assumed that the latter is predicted by the parent’s ineffective defenses against the psychic pain related to the abusive experiences they suffered in the past, above all identification with the aggressor. Ineffective defenses did not allow them to develop the ability to mentalize in close relationships, especially when painful or difficult emotions are at play, preventing abused parents from working on their traumatic experiences and thus mentalizing them. If they defended themselves by identifying with the aggressor, they would be at high-risk of negative caregiving, unable to stay emphatically in contact with themselves and with their children in times of distress. In their next paper, Fonagy et al. (1994) reported that all the mothers in the London Parent-Child Project with past experiences of trauma and deprivation who showed good reflective functioning had children that were classified as securely attached. A few empirical studies (Schechter et al., 2005; Huth-Bocks et al., 2014; Stacks et al., 2014) supported the notion that neither the severity of the traumatic events nor the severity of maternal post-traumatic stress disorder (PSTD) symptoms predicted the levels of maternal reflective functioning about the child. Several authors (for a review Allen, 2013) theorized that the ability to mentalize the experiences of abuse and neglect is the most important factor in the resilience process. Thus, it became crucial to empirically investigate parental reflective functioning in mothers who were abused in their childhood. Ensink et al. (2014) hypothesized that it is specifically the ability to mentalize one’s own past traumatic experiences rather than the mother’s general mentalizing capacity that prevents her from inadequate caregiving. In a sample of 100 pregnant women who had suffered from childhood abuse and neglect, they found that reflective functioning about the traumatic experiences was lower than the general mentalization about their childhood, and that low mentalization about the traumatic experiences was the best predictor of the difficulty to invest in the pregnancy as well as of the lack of positive expectations about becoming a mother. Ensink et al. (2014) also noted that abused mothers mostly had low reflective functioning and that more than one third of the mothers in their sample were classified on the AAI as unresolved regarding trauma.

These results have notable implications for prevention and intervention because it is well-known that unresolved mothers are more likely to have disorganized children and that infant attachment disorganization is a powerful predictor of later psychopathology (for a review, Madigan et al., 2006). Further support came from a recent study (Berthelot et al., 2015) that found that children of abused and neglected mothers were classified as insecure in 83% of cases and disorganized in almost half of the cases, with high concordance between maternal and child attachment. Furthermore, they found that maternal unresolved state of mind regarding the trauma and low reflective functioning about the trauma independently contributed to infant disorganization of attachment. Taken together, these studies call for the planning of appropriate clinical intervention designed to improve parental reflective functioning especially in high-risk mothers who show lower parental reflective functioning during pregnancy when compared to low-risk women (Smaling et al., 2015).

### Mentalization-Based Clinical Interventions

Mentalization-based interventions were designed to overcome the limitations that behavioral and psychoeducational parenting interventions showed in improving caregiving behaviors (for a review, see Suchman et al., 2006). In the present review only empirical studies investigating the improvement of the parental reflective functioning as an outcome of mentalization-based treatment were taken into account. Examining these studies is important not only to establish and to improve prevention and clinical interventions in favor of children and their parents, but also to shed light on issues of causality regarding parental RF and quality of caregiving behavior. In fact, the previously reviewed correlational studies did not demonstrate a causal relationship in that parental RF is what actually promotes good parenting competency. Thus, investigating whether the caregiving behavior improves as a result of the improvement of parental RF after a mentalization-based treatment might provide some basis for a causal relationship.

Pajulo et al. (2006) developed a residential program for mothers who suffered from substance abuse (alcohol and other drugs) and their babies. The treatment, which was carried out by units made up of social workers, psychologists, counselors, and occupational therapists, took place every day for approximately a 6-month period from the third trimester of pregnancy. In addition to providing an intervention that focuses on the general needs of the mothers and the children, the staff were specifically trained to focus on improving the mothers’ mentalization about themselves, their child and their relationship with the child. In particular, in an effort to prevent the mothers from returning to substance abuse, staff members helped them deal with challenging and painful feelings by sharing and discussing these issues. Videotaping some mother-child interactions and later reflecting upon them with the mothers was a further instrument that was used in the program to improve maternal reflective functioning. A recent outcome study (Pajulo et al., 2012) reported that RF levels increased from pregnancy (measured through PI-RF) to the post-natal phase (assessed by PDI-RF) in the majority (63%) of mothers who received the treatment. Mothers who abused alcohol and who reported more severe post-traumatic experiences showed a lower increase of RF. Furthermore, mothers whose children were later placed into foster care had lower RF, both during pregnancy and in the post-natal phase. The major limitation of this study was not having used a randomized controlled design, which was instead used in the following studies we reviewed.

Suchman et al. (2008, 2010a,b) developed the Mother and Toddler Program (MTP) in an effort to enhance the maternal ability to be sensitive and responsive to child communications in substance-abusing mothers with children up to 3 years of age. The program, consisting of 12 weekly sessions of individual therapy, went through the formation of the therapeutic alliance, the engagement of the mothers in mentalizing about the stressful situations associated with the parenting, and in dealing with the mental representations of the child and the relationship. The aims were to make the mother aware of the emotionally painful and stressful situations she experienced with the child which triggered her reaction to become disengaged from the child and/or to distort and misinterpret the child’s needs and communication. Finally, the sessions focused on mentalizing the child. In the MTP, therapists and mothers also watched some videotaped mother-child interactions together in order to encourage the mother to reflect on her own and on her child’s emotional and mental states. In addition, the MTP included guidance about the child’s psychosocial development at different ages. A randomized controlled trial was conducted to test the efficacy of the MTP versus the Parent Education Program (PE) which provided individual case management and child guidance pamphlets. The MTP was conducted by individual therapists while the PE was carried out by counselors. Both treatments were associated with comprehensive care including a great array of interventions, such as group cognitive-behavioral therapy, pharmacological therapy, vocational counseling, and child care. At post-treatment assessment, mothers enrolled in the MTP showed higher increases in PDI-RF as well as in representation quality of the relationship with their child and in caregiving behavior (Suchman et al., 2010b). Their next study (Suchman et al., 2011) examined which components of maternal reflective functioning improved after the MTP. Results showed that at post-treatment only self-focused RF, and not child-focused RF, significantly improved in mothers who took part in the MTP compared to women who followed the PE. The improvement was maintained, although to a lesser extent, even after a 6-week follow-up. These results appeared to be in line with the finding that the MTP focused primarily on mothers’ mentalizing about their struggle in regulating their own challenging emotional states and their effect on the child. At the 6-week follow-up visit, mothers enrolled in the MTP showed a more adequate caregiving behavior and their children were more able to communicate with them. A further study (Suchman et al., 2012) demonstrated that of all the components of the treatment, the therapist’s adherence to the MTP component which focuses on mentalization was what actually improved the mothers’ reflective functioning and their mental representation of the relationship with their children, the latter of which was the only factor that actually led to an improvement in caregiving behavior.

Baradon et al. (2008) developed a short-term, attachment-based group-intervention named “New Beginnings” specifically designed for mothers in prison with their babies. The manualized group-intervention focused on the child’s attachment needs by helping the mothers to mirror the child’s emotional states and to become more able to reflect and to talk about the child’s emotional experience, as well as to engage in attuned interactions with the child, thus increasing the maternal ability to provide contingent responses to their infants. The primary aim of the intervention was to promote a secure attachment pattern in the child in order to break the cycle of insecurity and the disorganization of the attachment. Helping the mothers to take a psychological stance regarding themselves, their child, and their relationship with the infant, as well as discussing topics regarding past and present ways of experiencing themselves and their relationships, and by making links with their present relationship with the child might increase the maternal ability to mentalize, which, in turn, would promote the development of a secure attachment pattern in the child. Each New Beginnings group was conducted by an experienced psychodynamic psychotherapist and included up to six mothers and their infants. It lasted four consecutive weeks, with 2 two-hour sessions weekly. Each session dealt with a topic that was chosen to activate the attachment system, such as pregnancy, maternal representations of their childhood history, their ideas about the child, their experience of being a mother, and their hopes about their own as well as their child’s future. Some session time was dedicated to playing with the children and then to talking together about the child’s communication in order to enhance maternal reflective functioning. In addition, mothers enrolled in the program were given handouts, spreadsheets, and homework. Mothers were invited to collect all the material in a folder, including their drawings, poetry or letters, in order to hold the memory of their experience during the program within themselves and to keep it for the future for the child. In addition, sharing the important emotional experiences with other mothers could foster their sense of belonging to a group which served as a secure base and provided its beneficial effect even after the end of the program. A comparison between pre-and post-treatment RF-PDI scores showed a significant increase in reflective functioning after the group-intervention. In particular, mothers showed an increase in reflective functioning when responding to the PDI questions regarding the recall of a positive experience with the child and concerning the child’s feelings about being rejected by the mother (Baradon et al., 2008). A following study (Sleed et al., 2013) used a cluster randomized controlled trial to test the efficacy of the New Beginnings; a group of mothers in prisons where group-intervention was provided was compared to a group of mothers residing in prisons where New Beginnings was not available. Results revealed that only mothers who took part in the group interventions showed a statistically significant improvement in reflective functioning, whereas mothers who did not receive the New Beginnings intervention showed a significant decrease in mentalizing both themselves as parents and their child. Mothers in the New Beginnings Program also showed some improvements in their interaction with the child, but the degree of change in reflective functioning did not significantly correlate with the degree of change in the mother-child interaction. Sleed et al. (2013) hypothesized that changes in behavior and changes in maternal mentalizing occur with different timing, thus they stated the need for further longitudinal studies in order to carry out a more in-depth investigation into the process of change at a representational as well as at a behavioral level.

Sadler et al. (2006) implemented an interdisciplinary, home-visiting program named Minding the Baby (MTB) which integrated and elaborated two evidence-based programs, namely the Nurse-Family Partnership (NFP; Olds, 2006) and infant-parent psychotherapy (IPP; Lieberman et al., 1999). The Minding the Baby intervention is conducted by nurses and mental health professionals (usually social workers) with post-graduate training who alternate in visiting the mothers once a week from the third trimester of pregnancy till the child’s first birthday, and then once every 2 weeks until the child’s second birthday. The nurses and the social workers follow the manualized guidelines in carrying out the intervention. Although nurses mostly focus on prenatal care and health education while social workers are more devoted to mental health and psychological issues regarding both mothers and children, both health professionals support reflective functioning using specific techniques to improve the maternal capacity to reflect upon their mental states as well as the child’s feelings and emotional needs (for an in-depth description of the MTB intervention, see Sadler et al., 2006). Among other outcomes, a cluster randomized controlled trial (Sadler et al., 2013) investigated the improvement in reflective functioning in mothers who received the MTB intervention and in mothers who received the standard care at their local community health center when the children were 2 years old. All the participants in the study were first-time mothers aged 14–25 years and none used heroin or cocaine, or had psychotic disorders or major medical conditions. Maternal RF was measured through the PI when mothers were in the third trimester of pregnancy and it was again assessed by administering the PDI when the children were 2 years old. Results showed that maternal RF improved in both groups without significant differences between mothers who received the MTB intervention and those who did not. Conversely, children whose mothers received the intervention were significantly more securely attached and less disorganized, thus the unexpected finding was specifically related to the maternal reflective functioning which did not improve specifically in the intervention group. A significantly different improvement in maternal reflective functioning was found only in mothers in the intervention group who had absent or very low RF before the birth of the child (PI-RF < 3). This finding raised in Sadler and co-workers some questions about the appropriateness of the RFS for assessing maternal mentalization in a sample of young and poorly educated women. They argued that RF score is very dependent on language skills and that mothers in their sample, who were mostly bilingual and with limited education, could have found it very challenging to use language to communicate and describe emotional and cognitive states. In addition, they highlighted that maternal RF develops not only during the 1st years of life, but also throughout adolescence, thus they argued that the young age of the mothers in their sample could have contributed to their unexpected findings. The first follow-up study, conducted 1–3 years after the intervention, showed that mothers who received the MTB treatment reported significantly fewer externalizing behaviors in their children, despite not reporting higher PDI-RF (Ordway et al., 2014).

A recent, randomized controlled trial (Fonagy et al., 2016) which investigated, among other outcomes, the change in maternal RF, yielded similar results and found no significant increase in maternal PDI-RF when mothers who received psychoanalytic PIP which strongly focused on the mothers’ mentalizing, were compared to mothers who only underwent standard intervention. Fonagy and co-workers also raised doubts about the lack of sensitivity of the PDI-RF due to its reliance on the use of mental-state language. In fact, they observed that their sample consisted of distressed mothers who were voluntarily seeking psychological support and that, unlike the Sadler et al. (2013) cohort, these women used a great deal of mental-state talk during the PDI transcripts, indicating preoccupation about emotional states rather than genuine mentalizing ability. Mental-state talk led to assigning an RF score, even though it did not qualify *per se* for a high RF score, therefore, it could be difficult to distinguish when the RF score indicates a real ability to mentalize and when it is related to the use of preoccupied mental-state talk.

Interestingly, to overcome this issue, Fonagy and co-workers used a qualitative measure named Assessment of Representational Risk (ARR; Sleed, 2013, Unpublished) to evaluate PDI transcripts, according to which the contents of the parents’ representations of their child as well as of their parenting are assessed according to the following subscales: Hostile, Helpless, and Narcissistic representations. In their study, only mothers in the PIP group showed a significant decrease in representational risk, particularly in the Helplessness and in the Hostile subscales. Fonagy and co-workers interpreted this finding as supporting the efficacy of the PIP intervention in changing the mothers’ representations of their child and specifically reducing feelings of helplessness and hostility toward their child.

The sole randomized controlled trial that investigated parental reflective functioning in the parents of children suffering from a neurodevelopmental disorder found a significant improvement in parents who received a 12-week relationship-based intervention when compared to the parents in the psychoeducational (wait-list) group (Sealy and Glovinsky, 2016).

In summary, although most of the reviewed studies showed that mentalization-based clinical interventions were effective in improving maternal mentalization and quality of caregiving, some studies raised issues which need further investigation. Particularly, (a) longitudinal studies are needed to study the process of change both at a representational and behavioral level with regard to the relationship between the two levels and their timing, and (b) further studies would be useful to investigate the influence of linguistic abilities and propensity to use mental-state talk on PDI-RF scores, and to explore parental mentalizing by using alternative measures. Taken together, the studies currently offer partial support in favor of a causal relationship between parental RF and adequate parenting.

### The Parental Reflective Functioning Questionnaire

The most recent studies introduced a new measure of parental RF, i.e., the Parental Reflective Functioning Questionnaire (PRFQ; Luyten et al., 2009, Unpublished). Although to date the developers of the PRFQ have not published the validation study, the measure, which is available from the authors upon request, has been used in a few empirical studies that herein will be briefly reviewed.

Rutherford et al. (2013) described the PRFQ as an 18-item questionnaire developed to overcome the limitations of very time-consuming interview-measures. Items relate to pre-mentalizing modes (e.g., “When my child is fussy he or she does that just to annoy me”), to the degree of the certainty of mental states as opposed to the awareness of the opacity of the mental states (e.g., “I always know why my child acts the way he or she does”), and to the level of parental interest and curiosity in mental states (e.g., “I am often curious to find out how my child feels”). Each item receives a score on the 7-point Likert scale, from “1” to express “strongly disagree” to “7” to indicate “strongly agree.” Rutherford et al. (2013) reported that in the not yet published validation study (Luyten et al., 2009, Unpublished), the three-factor structure of the PRFQ was supported by exploratory and confirmatory factor analysis in two different samples for both mothers and fathers, and that internal consistency was good for all the subscales, ranging from 0.70 for pre-mentalizing modes to 0.82 for certainty in mental states. The subscales were not correlated with demographic features, whereas they were associated with parental attachment, emotional availability, and parenting stress.

Bottos and Nilsen (2014) found that if associated with past experiences of emotional maltreatment, maternal impairment in RF as assessed by PRFQ mediated the detrimental effect of maternal depression on the child’s mentalizing abilities. Rostad and Whitaker (2016) found that parental interest and curiosity in mental states as assessed by PRFQ were associated with the degree of satisfaction with parenting, involvement, and communication. Rutherford et al. (2013, 2015) found that parental reflective functioning evaluated by PRFQ was related to the mother’s tolerance of infant distress. In their next study, Rutherford et al. (2016) demonstrated that PRFQ scores were associated with the neural correlates of infant cue perception, showing an association between PRFQ scores and parental sensitivity to the child’s emotional signals. PRFQ was also used in two studies aimed at evaluating the efficacy of the intervention. Ashton et al. (2016) found an improvement in parental reflective functioning in mothers who underwent intensive group intervention (Trauma and Attachment Group; TAG) for caregiver/child dyads. On the contrary, in a sample of substance-abusing mothers, Paris et al. (2015) reported that PRFQ scores revealed a significant improvement only in mothers who had shown higher levels of psychological distress at the beginning of a brief dyadic parent-child intervention. A prenatal version (Prenatal Parental Reflective Functioning Questionnaire; P-PRFQ) consisting of 14 items was recently developed by Pajulo et al. (2015). The initial validation study showed a three-factor structure (F1: “Opacity of mental states”; F2: “Reflecting on the fetus-child”; and F3: “The dynamic nature of the mental states”) and suggested it could be a valid and promising new instrument for assessing parental reflective functioning during pregnancy.

Although it is too early to evaluate the new, recently developed measure, the reviewed studies appear to be promising. The measure could potentially overcome some critical issues that have come up because of the use of narrative interviews that necessarily rely on linguistic abilities and verbal patterns, and furthermore, it would appear to be much easier to use and less time-consuming. These qualities could make the measure very valuable for screening and large scale-studies.

## Discussion and Direction for Future Research

The reviewed studies support the notion that parental RF is associated with adequate caregiving and the child’s attachment security. Low maternal RF was found in mothers whose children suffered from anxiety disorders (Esbjørn et al., 2013), impairment in emotion regulation (Heron-Delaney et al., 2016), and externalizing behaviors (Ensink et al., 2016a; Smaling et al., 2016b). Moreover, higher parental RF was associated with better mentalizing abilities in children (Benbassat and Priel, 2012; Ensink et al., 2015; Rosso et al., 2015; Scopesi et al., 2015).

Since a vast array of studies have demonstrated that the cycle of abuse can come to an end if abused mothers are able to mentalize their past abusive experiences, mentalization-based interventions were developed to help mothers at high risk of repeating the abusive parenting. Taken together, the reviewed studies found a significant improvement in maternal caregiving after the treatment and most of the studies also yielded a significant improvement in maternal RF in the mothers who received mentalization-based interventions. However, two recent studies (Sadler et al., 2013; Fonagy et al., 2016) found a general improvement in the mother-child relationship, but quite notably they did not find a significant increase in PDI-RF when comparing the outcomes of mentalization-based treatment with the outcome of other interventions. Sadler et al. (2013) raised doubts about the appropriateness of the PDI-RF because its scoring is highly dependent on language, and this could be a bias in studies conducted on high risk samples of poorly educated mothers. Conversely, Fonagy et al. (2016) questioned the fact that PDI-RF scoring is inevitably influenced by the mental state talk produced in the PDI narrative. In fact, extensive use of mental state talk inevitably led to assigning an RF score of at least 3, even though the use of mental-state talk is not always indicative of a real ability to mentalize. They found a great deal of mental state talk in the PDI narratives produced by the mothers asking for psychological support, but they observed that it was not a marker of an authentic ability to mentalize. It could be hypothesized that preoccupied mothers make a more extensive use of mental state talk, although they are not really able to mentalize.

In order to overcome the limitations of PDI-RF, two further measures of parental RF were recently developed: a content analysis of the maternal representations in the PDI transcripts (ARR; Sleed, 2013, Unpublished) and an 18-item questionnaire (PRFQ; Luyten et al., 2009, Unpublished). To date, the AAR has only been used in two studies, whereas the PRFQ has been used in several studies in the last 3 years thus proving to be a promising measure of general parental reflective functioning, which we assume could be used as a reliable screening instrument.

Several questions remain unanswered, thus further investigation is needed. Firstly, the role of paternal reflective functioning on the child’s development has been neglected altogether. Paternal RF was investigated by Stover and Kiselica (2014) alone in a mixed sample of fathers (half of whom had substance-abuse and violence problems, and half who did not). They found an association between lower PDI-RF, poorer education, and substance-abuse, whereas a correlation between PDI-RF and parenting behavior, as investigated by self-report measures, was not found.

Secondly, surprisingly no published studies are available regarding the association between AAI-RF and PDI-RF. We suggest that it might be relevant to investigate to what extent the maternal ability to mentalize her own childhood attachment experiences is associated with her ability to mentalize in the context of the relationship with her child. In addition, such studies would allow us to investigate how maternal attachment patterns impact on the use of mental state talk. We hypothesize that preoccupied mothers might make a great use of mental state talk, thus researchers should not only take into account PDI-RF but the attachment pattern as well when evaluating the quality of the mental state talk.

Thirdly, further studies are needed to investigate the psychometric properties of the PDI-RF, especially regarding the impact of I.Q., linguistic competence, education, and the child’s age on scoring. Two studies alone (Suchman et al., 2010a; Borelli et al., 2016) investigated the factor structure of the PDI-RF, identifying two factors, namely self-mentalization and child-mentalization. To date, the contribution of each of the two identified PDI factors on the quality of the mother-child relationship as well as on the child’s psychological development has been insufficiently investigated. Further studies are needed to replicate and to investigate more in-depth the important findings by Suchman et al. (2010a) which support the notion that it is the maternal ability to deal with her own emotional experience elicited by the relationship with her child that allows her to respond sensitively to her child’s emotional needs.

Fourthly, in our opinion some components of the parental reflective functioning, such as the painful affect mentalization versus non-painful affect mentalization, deserve more in-depth investigation since some studies (Fonagy et al., 2002; Sharp et al., 2006; Borelli et al., 2012; Rosso et al., 2015) demonstrated that the maternal ability to mentalize painful emotional experiences is especially crucial for the child’s development.

Finally, and most importantly, we propose that it is highly necessary to investigate whether parental mentalization can really be detected through verbal measures, such as the ones we took into account in this review. Some years ago, Shai and Belsky (2011) stated that sole reliance on verbal processes may be unsuccessful in capturing mentalizing processes, thus they introduced the notion of parental embodied mentalizing, which refers to the parental ability to implicitly understand the child’s mental states through all the movements of her/his body and to attune with them through their own kinesthetic patterns. Thus, they hypothesized that it is this form of unconscious mentalization that needs to be investigated in order to study the parental capacity to be adequately responsive to the child’s emotional needs. We argue that the unexpected findings from the most recent randomized controlled trials reviewed in the current paper (Sadler et al., 2013; Ordway et al., 2014; Fonagy et al., 2016) could be in line with Shai and Belsky’s (2011) formulations.

## Conclusion

Taken together the reviewed studies offer strong support to the determinant influence of parental reflective functioning on the quality of caregiving, on the child’s attachment security, on the child’s emotion regulation, and on the child’s reflective functioning. Randomized controlled trials showed that mentalization-based interventions were effective in improving caregiving, which is highly relevant especially regarding mothers who have a history of maltreatment and thus who are at high risk of becoming maltreating parents. Nevertheless, some issues require further investigation. In particular, the role of paternal reflective functioning on the child’s development, the association between the ability to mentalize one’s own childhood experiences and the ability to mentalize the child and the relationship with her/him, as well as the diverse role of the parental ability to mentalize painful versus non-painful emotions should be investigated more thoroughly. Lastly, additional studies are needed to investigate the psychometric properties of PDI-RF both regarding its factor-structure and its sensitivity in detecting reflective functioning because its scoring is dependent on linguistic skills and the propensity to use mental state talk. In addition, studies on the development of non-verbal measures of parental mentalization are warranted.

## Author Contributions

The author confirms being the sole contributor of this work and approved it for publication.

## Conflict of Interest Statement

The author declares that the research was conducted in the absence of any commercial or financial relationships that could be construed as a potential conflict of interest.
